# Assessing the causes for the dentistry selection as a job by Iranian dental students: A Questionnaire Survey

**Published:** 2015

**Authors:** J Hamissi, P Bairami, Z Hamissi

**Affiliations:** * Department of Periodontics and Dental Caries Prevention Research Center, Qazvin University Medical Sciences, Qazvin, Iran,; ** Department of Dentistry, Al Ghadier General Hospital, Tabriz, Iran,; *** College of Dentistry, Shahied Behesti University of Medical Sciences, Teheran, Iran,

**Keywords:** dentistry, career, questionnaire, dental students, dental education

## Abstract

**Objective:** The purpose of the current research is to discover the causes for the dentistry selection as a profession by senior dental scholars in Iran.

**Materials & Methods:** Surveys are distributed between the first-year to the sixth-year senior dental scholars at the Scholl of Dentistry in Medical Sciences Qazvin University in Iran. The survey presented in a lecture hall at the terminal of the secondary term in the 2011-2012 academic years. The survey included 30 parts, and the pupils asked to measure the significance of any part for choosing dentistry as a profession, on a 10-points scale. In addition, T-test and ANOVA utilized for the data analysis.

**Results:** The answering ratio in the research is 55% (out of 100%) of the pupils delivered. Ninety-six students (93.2%) selected it as an initial selection. Dentistry as a job which is “insurer of financial independence” was gained a most rate via 82.5% of the pupils raised and a comparable amount of the students (75.7%), assigned a highest rate to the parameter “I love to enhance many money”. Dentistry as a “science-based job” was also given a score by 80.6% of the pupils.

**Conclusion:** There are no variations in the motivation between men and women students. It obtained that “insurer of financial independence” and “I love to enhance many money” are critical action parameters in the dental pupils population.

## Introduction

The motivations for selecting dentistry as a job in Iran are not clear. This study was aimed at exploring the causes why students chose this career. Dentists have an important position in the society as licensed healthcare workers [**1**,**2**].

Dentistry is ranked fourth in comparison with medicine, pharmacy, and veterinary science. Consequently, an academic activities high level of is necessary to start a job as a dentist. But, fewer obviously expressed are the parameters that impact the dentistry selection as a job. Ordering and correlating these parameters would be useful in specifying the pupil’s requirement and requests in their undergraduate careers. Previous researches conducted elsewhere [**3**-**8**].

The considerations for taking a profession are complicated and a dentistry choice as a job is such as them. There were some factors deciding the career choices, such as: job status and financial and economic opportunities, the nature of the career, relation and work with community, etc. [**9**,**10**].

Taking dentistry as a profession has been investigated in most zones. A variety of causes were referred for this, involving: situation and policy, the career nature, cases care, and interfacing with community [**9**]. Majority of studies examining the ideas for a dentistry selection as a job have been conducted and investigated in advanced nations like the US [**10**], United Kingdom [**9**-**11**], Australia [**5**], Denmark [**14**] and Ireland [**12**]. Self-employment and business-related motives were reported as important factors by the students in the US [**10**] and perceived as easy of official working were becoming self-job, operation for normal times, and having a high money earn and the chance to assist community, being stated as the causes for entering this job in Ireland [**12**].

In Iran, the dental program lasts for at least for 6 years. Admission to the plan related to the completion of the Iranian University Entrance Exam (the Concurs) which requires 12 years of investigation in order to be qualified to take this exam. A great stage of educational achievement or a great-mark standard is vital for dental pupils to start a job as a dentist. There was restricted data considering the job selection of pupils in advanced zones [**15**].

Nevertheless, there is no available data regarding the causes for the dentistry selection as a job in the Iranian population. Our study is therefore aimed at investigating the carrier motivation.

## Method 

***Questionnaire***

The dental plan involves of 2 years of primary science hypothesis and one year of preclinical hypothesis and a three-year clinical phase. The approach regulated is an anonymous survey distributed by the interviwers to the first-year to the sixth-year pupils at the Dentistry Scholl in Medical Sciences Qazvin University. Moral support was awarded via the Medical Sciences Qazvin University. All partners get a survey copy prior the lecture was started and they were notified, within a concise oral communication, regarding the formation and the purposes of this study. During the completion of the survey, cases were requested to encounter some duties or issues that may have seemed unintelligible, as the review proposed at evaluating multiple planes of provision, from basically to highly ahead levels. The predictable time for the completion of the survey was ten minutes. The information gathered at the terminal of the second semester in the 2011-2012 academic years.

The three-page questionnaire utilized in this research was according to the previous studies [**10**,**12**,**15**]. In addition to the matters compared to social and house connections and the school selection methods, socio-demographic parameters like sexuality, community, household earnings, and parents’ profession listed. Furthermore, ideas that motivated dental scholars in their selection of the examination designated as well. These introduced the ideas characterized as “economically”, “people” and “business ( compliance )” oriented (**Table 1**). The survey turned to Persian to assure that the pupils clearly found all the aspects. It contains thirty objects with each object on the survey including a description that expected the respondents to dispense their approval grade on a 10-point rate varying from zero up to ten. 2 further inquiries responded via no or yes depending on the fact whether dentistry was the pupils original selection and if the pupil would choose dentistry repeatedly. There is further the chance to make free comments on the questionnaire.

**Table 1:** Wording of 30 items in the survey

*Factor*
1- A person or extra of my relations are dentists.
2- A person or extra my associates are dentists.
3- My Grade inspired me to take dentistry as an occupation.
4- I took dentistry since people influencing.
5- It is comfortable for D.D.S to get a profession.
6- Dentistry gives greater than other career opportunities apparent for me.
7- I desire to be self-working.
8- I desire to attend / assist bodies in improving their image.
9- Dentistry has more regular hours than other caring professions.
10- Other bodies prompted me to enhance a dentist.
11- I perpetually desired to be a dentist.
12- I can begin training dentistry separately after finishing.
13- I love working via characters.
14- I have a real skill while attending the house dentist.
15- I relish the liberty of the D.D.S.
16- An occupation in dentistry allows a better job safety.
17- A dentist had a more flexible plan.
18- Dentistry lets me have much time to be with family.
19- Choosing dentistry provides the chance to do by my own hands.
20- It is a caring occupation.
21- It is an occupation according to science.
22- I love to earn many incomes.
23- It is a career via more prestige.
24- I heard regarding it in high school.
25- I heard regarding it in university.
26- The relative’s dentist inspired.
27- Dentistry as a job assures economic freedom.
28- There are not “on-call” task.
29- Dentists ordinarily do not cope with lives (death and life) condition on a conventional system.
30- I accomplished in a relevant dental area, and I desire to be.

**Table 2:** Answers ratio

Year	No.	Percentage
1	15	14.9
2	18	17.8
3	27	26.7
4	23	22.8
5	18	17.8
Total	101	100

**Table 3:** Head ten causes for seeking a profession in dentistry for men and women, as defined by average degree

No.	Factors	Scores (Mean)	(Sd)
	*Male*		
13	I like working with people.	(4.28)	(3.52)
21	It is a science-based profession.	(4.15)	(3.62)
27	It is as a job assures economic ability.	(4.05)	(3.35)
8	I desire to treat cases to enhance their appearance.	(4.03)	(3.21)
22	I love to earn many money.	(3.93)	(3.4)
16	A job in dentistry lets better safety.	(3.88)	(3.44)
15	I want the freedom of dentists.	(3.78)	(3.6)
23	It is a prestigious job.	(3.77)	(3.36)
18	Being dentist let me more time to answer via my family.	(3.75)	(3.39)
20	It is a healthcare job.	(3.52)	(3.23)
	*Female*		
27	Dentistry as a career ensures economical freedom.	(3.77)	(3.24)
10	Additional characters inspired me to be a dentist.	(3.48)	(3.41)
29	D.D.S ordinarily do not cope with life-or-death situations on usual.	(3.47)	(3.56)
15	I like the autonomy that dentists have.	(3.43)	(3.27)
3	My grade inspired me to select dentistry as an occupation.	(3.37)	(3.48)
22	I like to make a lot of money.	(3.35)	(3)
7	I desire self-employment.	(3.35)	(3.95)
23	It has more authority.	(3.31)	(3.11)
14	I have a well skill while visiting the friend’s dentist.	(3.29)	(3.61)
5	Easy for dentists to get job.	(3.27)	(3.05)

**Table 4:** Parameter loadings on fundamental motive parameters

Factors	Parameter 1	Parameter 2	Parameter 3
	Money	People	Flexibility
	Male	Male	Male
	Female	Female	Female
6-Dentistry earns more than other jobs.	3.48		
	3.26		
27-Dentistry as a job assures economic independence.	4.05		
	3.77		
5- It is easy for dentists to get job.	3.23		
	3.27		
7- I eager to be self-working.	2.1		
	3.35		
22- I like to make a lot of money.	3.93		
	3.35		
16- A job in dentistry provides better safety.	3.88		
	3.26		
15- I love the authority and prestigious of dentists.	3.78		
	3.43		
29- Dentists normally do not cope with life-or-death situations on a usual.		3.2	
		3.47	
8- I desire to behave cases to enhance their face.		4.03	
		3.18	
13-I like working with people.		4.28	
		2.88	
19- It gives me the chance to act via my own hands.		3	
		3.13	
18- Dentistry let me more time to have via family.			3.75
			3.03
28- There was not much “un-call” task.			3.45
			3.11
29- Dentists normally do not cope with death conditions on a usual.			3.2
			3.47
9- Dentistry has more regular hours than other caring professions.			2.88
			2.19
17- Dentistry has a flexible plan.			3.2
			2.87
Mean (total)	3.49	3.63	3.05
	3.31	3.17	2.37

**Statistical Analysis**

The returned surveys were checked for fulfilling of information; the information was concocted and examined using the SPSS. The t-test was applied to examine either statistically meaningful distinctions survived among rates for various teams. The t-test was applied for the investigation of the 2 teams and ANOVA was used for more than two groups. Grade means for men and women evaluated and a free case t-test is then utilized to investigate the variations in the rates compared to sec and the initial selection team and the non-first selection cluster.

## Results 

An overall response rate of 55% (103 out of 188) was obtained. Out of 103 respondents, 39.2% (40) were males and 60.8% (62) females, and one student did not recognize his/ her gender. There is no analytically clear distinct in gender distribution among the participants from each year (P>0.05). Iranian dental students can directly enter the Dental School after graduating from high school and after passing the National University Entrance Exam. At the investigation duration, the respondents age varied of 18 to 27, with a median 21 years old. 

The most of the pupils (93.2%) placed dentistry as their initial job selection. In other words, 3 percent of the pupils placed other courses, other than dentistry, as their first preferences. 

Most fathers (34.5%) were clerks and most mothers (59.8%) were housewives. Also, half of the fathers had a bachelor and 29.6% of the moms had higher degrees. This included 10.3% of the dads and 4.5% of the moms via postgraduate degrees. There is no clear and notable relation among the parents’ educational levels.

## Discussion

The educational requirements, admission requirements, and selection procedures greatly varied among schools and, not surprisingly, the effect of these requirements was showed in the sociodemographic profile of the pupils. Additionally, there is an enhance in female representation among the dental students. A similar trend has also been seen in the USA [**16**], the UK [**17**] and South Africa [**7**].

The majority of pupils (93.2%) placed dentistry as a initial career choice, and the majority of those who did not, had it as their second choice (65%). This represents an increase from the 43% stated to have placed dentistry as a first choice in the 1981-1985 period [**18**] and in the mid-1990s [**13**]. However, some research reports [**18**] found no distinct in the educational action or graduation rate of “first choice” pupils and other pupils. Still, this is identical to the almost 90% stated in the United Kingdom [**19**]. Qualitative approaches are obligatory to explore and understand the pupils causes for selecting or not selecting it as a job. Moreover, because of the limited professional and longitudinal studies on oral health, studies should survey the career path and retention according to career choices [**20**].

The findings of a research in the US showed that there are distinct in the motivation of men and women students [**10**]. Female students were few concerned than men pupils by the company part of a job choice (were less financially oriented) and were more related with the healthcare and people parameters (were more people (caring)-oriented) [**10**,**21**]. This was similar to a recent study conducted by the initial dental pupils in Peru [**14**].

However, in this research, the high number of the female students who have chosen dentistry as a first choice may be because of the easy dental practice requirements.

It can be get that “helping people” and “prestige” are main exciting parameters for this team of dental pupils [**15**]. Our study came up with the same results.

The findings proposed in the current investigation are the initial gathered and stated inforamtion in the Islamic Republic of Iran on the reasons and motives for selecting dentistry as a job. 

## Conclusion

The present study was the first information on the Iranian population, looking into the selection of dentistry as a job. “helping people” and “Prestige” realized to be critical excitation parameters for this team of dental pupils. There are furthers analytically clear distinct in the motivation between men and women students with the financial factors being more influential for the former. Of concern is the finding that 50% of the pupils indicated that dentistry is not their initial selection of job. It might be a proper opinion to establish job training plans in college in order to train pupils regarding their job selection and employment chances after graduation. According to the obtained findings, it was concluded that the causes for selecting dentistry as a professional job are distincts for the sexes. 

**Acknowledgements**

The authors want to thank the anonymous referees for their commentaries on an earlier draft of this paper. And also, we would like to acknowledge the participation of students without whom we would not have reached this high response rate. The authors are also greatly indebted to Professor Al-Bitar, Z.B from the University of Jordan for his helpful comments and contribution to the manuscript. 
